# Methyl eucomate

**DOI:** 10.1107/S1600536808018734

**Published:** 2008-06-28

**Authors:** Linglin Li, Guang-Xiong Zhou, Ren-Wang Jiang

**Affiliations:** a Nutrition and Metabolism Laboratory, Beth Israel Deaconess Medical Center, Boston, MA 02215, USA; b Institute of Traditional Chinese Medicine and Natural Products, College of Pharmacy, Jinan University, Guangzhou 510632, People’s Republic of China

## Abstract

The crystal structure of the title compound [systematic name: methyl 3-carboxy-3-hydr­oxy-3-(4-hydroxy­benz­yl)propanoate], C_12_H_14_O_6_, is stabilized by inter­molecular O—H⋯O and C—H⋯O hydrogen bonds. The mol­ecules are arranged in layers, parallel to (001), which are inter­connected by the O—H⋯O hydrogen bonds.

## Related literature

For related literature, see: Heller & Tamm (1974 ▶); Jiang *et al.* (2002 ▶, 2006 ▶).
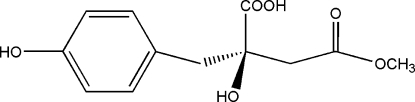


## Experimental

### 

#### Crystal data

C_12_H_14_O_6_
*M* *_r_* = 254.23
Orthorhombic, 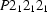

*a* = 5.9109 (6) Å
*b* = 7.0348 (7) Å
*c* = 29.109 (3) Å
*V* = 1210.4 (2) Å^3^
*Z* = 4
Mo *K*α radiation
μ = 0.11 mm^−1^
*T* = 293 (2) K
0.40 × 0.32 × 0.25 mm

#### Data collection

Bruker SMART CCD diffractometer
Absorption correction: none
6709 measured reflections
1279 independent reflections
1047 reflections with *I* > 2σ(*I*)
*R*_int_ = 0.045

#### Refinement

*R*[*F* ^2^ > 2σ(*F* ^2^)] = 0.029
*wR*(*F* ^2^) = 0.066
*S* = 1.05
1278 reflections
168 parameters
H-atom parameters constrained
Δρ_max_ = 0.14 e Å^−3^
Δρ_min_ = −0.12 e Å^−3^

### 

Data collection: *SMART* (Bruker, 1998 ▶); cell refinement: *SAINT* (Bruker, 1998 ▶); data reduction: *SAINT* and *XPREP* in *SHELXTL* (Sheldrick, 2008 ▶); program(s) used to solve structure: *SHELXS97* (Sheldrick, 2008 ▶); program(s) used to refine structure: *SHELXL97* (Sheldrick, 2008 ▶); molecular graphics: *XP* (Siemens, 1998 ▶); software used to prepare material for publication: *SHELXTL*.

## Supplementary Material

Crystal structure: contains datablocks global, I. DOI: 10.1107/S1600536808018734/fb2096sup1.cif

Structure factors: contains datablocks I. DOI: 10.1107/S1600536808018734/fb2096Isup2.hkl

Additional supplementary materials:  crystallographic information; 3D view; checkCIF report

## Figures and Tables

**Table 1:** Hydrogen-bond geometry (Å, °)

*D*—H⋯*A*	*D*—H	H⋯*A*	*D*⋯*A*	*D*—H⋯*A*
O1—H1⋯O3^i^	0.82	1.96	2.775 (2)	172
O2—H2⋯O1^ii^	0.82	2.33	2.888 (2)	125
O4—H4⋯O2^iii^	0.82	1.85	2.639 (2)	161
C12—H12*B*⋯O5^iv^	0.96	2.42	3.268 (4)	148

Symmetry codes: (i) 
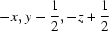
; (ii) 
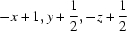
; (iii) 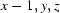; (iv) 
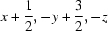
.

**Fig. 1.**
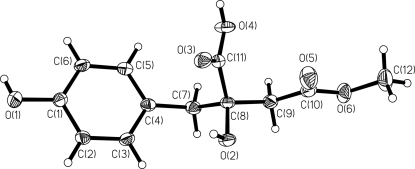
The molecular structure of the title structure showing 30% probability displacement ellipsoids and the atom-numbering scheme.

**Fig. 2.**
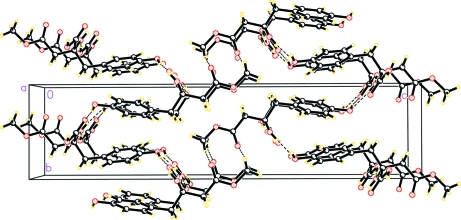
The packing diagram of the title structure viewed down the a axis.

## supplementary crystallographic information

### Comment

Methyl eucomate is the methyl ester of the eucomic acid. The title compound has been isolated from several edible plants, *e. g.* Opuntia dillenii (Jiang *et al.*, 2006) or Opuntia vulgaris (Jiang *et al.*, 2002). However, the stereochemistry of the ester has not been established yet. In the present paper, we report its crystal structure.

The molecule contains phenol, carboxyl, ester and hydroxyl functional groups (Fig. 1). The mean deviation of the benzene ring from planarity is 0.0004 Å and its dihedral angle with the plane of the carboxylic group at C8 is 50.3 (3)°, while it is roughly perpendicular to the ester group at C10 with a dihedral angle of 87.3 (3)°.

The intermolecular hydrogen bonds O1—H···O3, O2—H···O1 and O4—H···O2 (Tab. 1) link the molecules into layers that are parallel to (001) (Fig. 2).

There is no heavy atom with a significant anomalous dispersion contribution, so the absolute configuration from the diffraction pattern itself could not be determined.  However, the absolute configuration of the eucomic acid has been established by synthesis (Heller & Tamm, 1974) though its crystal structure has not been determined. Therefore the title compound is expected to share the same *R* configuration at the chiral centre C8.

### Experimental

The title compound was purified from the stems of Opuntia vulgaris according to the reported procedures (Jiang *et al.*, 2002). Briefly, the stems of Opuntia vulgaris (1 kg) was extracted with 95% ethanol under room temperature. The extracted solution was concentrated with rotary evaporator to afford a crude extract, which was suspended in distilled water and partitioned with petroleum ether, ethyl acetate and n-butanol. Then the n-butanol fraction was subjected to silica gel column chromatography eluted with methanol-chloroform gradient solvent system to afford the title compound (16 mg). The transparent rectangular crystals of the title compound with average size of 0.50 × 0.40 × 0.30 mm were obtained by slow evaporation of the methanol solution at room temperature.

### Refinement

Though all the hydrogens were discernible in the difference electron density maps. Neverheless, the hydrogens were situated into the idealized position and constrained during the refinement. Hydroxyl hydrogens: O-H equalled to 0.82 Å, U_iso_(H)=1.5 U_eq_O; C_aryl_-H equalled to 0.93 Å, U_iso_(H)=1.2 U_eq_C_aryl_; C_methylene_-H equalled to 0.97 Å, U_iso_(H)=1.2 U_eq_C_methylene_; C_methyl_-H equalled to 0.96 Å, U_iso_(H)=1.5 U_eq_C_methyl_.

There is no heavy atom with significant anomalous dispersion contribution in the structure for the used wavelength, so the absolute configuration from the diffraction pattern itself was not determined. 836 Friedel reflections were merged before the refinement. However, the absolute configuration of the related eucomic acid has been established previously (Heller & Tamm, 1974) and therefore the title compound has been expected to share the same *R* configuration at the chiral center C8.

Reflection (0 0 2) was omitted.

### Crystal data

**Table:**

C_12_H_14_O_6_	*F*_000_ = 536
*M**_r_* = 254.23	*D*_x_ = 1.395 Mg m^−^^3^
Orthorhombic, *P*2_1_2_1_2_1_	Mo *K*α radiation λ = 0.71073 Å
Hall symbol: P 2ac 2ab	Cell parameters from 6709 reflections
*a* = 5.9109 (6) Å	θ = 1.4–25.0º
*b* = 7.0348 (7) Å	µ = 0.11 mm^−^^1^
*c* = 29.109 (3) Å	*T* = 293 (2) K
*V* = 1210.4 (2) Å^3^	Rectangular, colourless
*Z* = 4	0.40 × 0.32 × 0.25 mm

### Data collection

**Table:**

Bruker SMART/CCD diffractometer	1047 reflections with *I* > 2σ(*I*)
Radiation source: fine-focus sealed tube	*R*_int_ = 0.046
Monochromator: graphite	θ_max_ = 25.0º
*T* = 293(2) K	θ_min_ = 1.4º
ω scans	*h* = −6→7
Absorption correction: none	*k* = −8→7
6709 measured reflections	*l* = −27→34
1279 independent reflections	

### Refinement

**Table:**

Refinement on *F*^2^	Hydrogen site location: difference Fourier map
Least-squares matrix: full	H-atom parameters constrained
*R*[*F*^2^ > 2σ(*F*^2^)] = 0.029	*w* = 1/[σ^2^(*F*_o_^2^) + (0.0328*P*)^2^] where *P* = (*F*_o_^2^ + 2*F*_c_^2^)/3
*wR*(*F*^2^) = 0.066	(Δ/σ)_max_ < 0.001
*S* = 1.05	Δρ_max_ = 0.14 e Å^−^^3^
1278 reflections	Δρ_min_ = −0.12 e Å^−^^3^
168 parameters	Extinction correction: SHELXL97 (Sheldrick, 2008), Fc^*^=kFc[1+0.001xFc^2^λ^3^/sin(2θ)]^-1/4^
42 constraints	Extinction coefficient: 0.0070 (19)
Primary atom site location: structure-invariant direct methods	Absolute structure:
Secondary atom site location: difference Fourier map	

### Special details

**Table:**

Geometry. All e.s.d.'s (except the e.s.d. in the dihedral angle between two l.s. planes) are estimated using the full covariance matrix. The cell e.s.d.'s are taken into account individually in the estimation of e.s.d.'s in distances, angles and torsion angles; correlations between e.s.d.'s in cell parameters are only used when they are defined by crystal symmetry. An approximate (isotropic) treatment of cell e.s.d.'s is used for estimating e.s.d.'s involving l.s. planes.
Refinement. Refinement of *F*^2^ against ALL reflections. The weighted *R*-factor *wR* and goodness of fit *S* are based on *F*^2^, conventional *R*-factors *R* are based on *F*, with *F* set to zero for negative *F*^2^. The threshold expression of *F*^2^ > σ(*F*^2^) is used only for calculating *R*-factors(gt) *etc*. and is not relevant to the choice of reflections for refinement. *R*-factors based on *F*^2^ are statistically about twice as large as those based on *F*, and *R*- factors based on ALL data will be even larger.

### Fractional atomic coordinates and isotropic or equivalent isotropic displacement parameters (Å^2^)

**Table:**

	*x*	*y*	*z*	*U*_iso_*/*U*_eq_	
O1	0.2505 (3)	0.2830 (2)	0.32392 (5)	0.0466 (5)	
H1	0.1313	0.2346	0.3323	0.070*	
O2	0.5510 (2)	0.5081 (2)	0.11441 (5)	0.0400 (4)	
H2	0.5099	0.5865	0.1335	0.060*	
O3	0.1405 (3)	0.6278 (2)	0.13901 (5)	0.0425 (4)	
O4	−0.0301 (3)	0.3948 (3)	0.09911 (6)	0.0499 (5)	
H4	−0.1460	0.4500	0.1070	0.075*	
O5	0.2745 (4)	0.6569 (3)	0.03265 (6)	0.0640 (6)	
O6	0.3709 (3)	0.4543 (2)	−0.02286 (5)	0.0543 (5)	
C1	0.2722 (4)	0.2631 (3)	0.27718 (8)	0.0344 (6)	
C2	0.4773 (4)	0.3133 (3)	0.25799 (8)	0.0367 (6)	
H2B	0.5932	0.3598	0.2764	0.044*	
C3	0.5089 (4)	0.2940 (3)	0.21133 (8)	0.0370 (6)	
H3A	0.6475	0.3286	0.1987	0.044*	
C4	0.3420 (4)	0.2253 (3)	0.18265 (8)	0.0346 (6)	
C5	0.1367 (4)	0.1752 (3)	0.20275 (8)	0.0389 (6)	
H5A	0.0209	0.1285	0.1843	0.047*	
C6	0.1010 (4)	0.1933 (3)	0.24947 (9)	0.0387 (6)	
H6A	−0.0373	0.1588	0.2622	0.046*	
C7	0.3804 (4)	0.2011 (3)	0.13179 (8)	0.0400 (6)	
H7A	0.2693	0.1119	0.1201	0.048*	
H7B	0.5286	0.1451	0.1272	0.048*	
C8	0.3660 (4)	0.3855 (3)	0.10324 (7)	0.0336 (5)	
C9	0.3831 (4)	0.3384 (3)	0.05217 (8)	0.0403 (6)	
H9A	0.5339	0.2904	0.0459	0.048*	
H9B	0.2767	0.2375	0.0452	0.048*	
C10	0.3374 (4)	0.5023 (4)	0.02090 (8)	0.0424 (6)	
C11	0.1476 (4)	0.4865 (4)	0.11534 (7)	0.0347 (5)	
C12	0.3196 (5)	0.5985 (4)	−0.05667 (9)	0.0681 (9)	
H12A	0.3552	0.5515	−0.0868	0.102*	
H12B	0.4079	0.7102	−0.0505	0.102*	
H12C	0.1617	0.6297	−0.0552	0.102*	

### Atomic displacement parameters (Å^2^)

**Table:**

	*U*^11^	*U*^22^	*U*^33^	*U*^12^	*U*^13^	*U*^23^
O1	0.0418 (10)	0.0604 (11)	0.0377 (10)	−0.0079 (10)	0.0050 (8)	0.0063 (9)
O2	0.0255 (9)	0.0527 (11)	0.0418 (11)	−0.0021 (8)	0.0013 (7)	−0.0105 (9)
O3	0.0368 (9)	0.0474 (10)	0.0432 (10)	0.0061 (9)	0.0051 (8)	−0.0103 (8)
O4	0.0229 (9)	0.0617 (12)	0.0651 (12)	0.0047 (9)	−0.0011 (9)	−0.0157 (11)
O5	0.0819 (15)	0.0646 (12)	0.0454 (11)	0.0208 (12)	0.0057 (11)	−0.0017 (10)
O6	0.0678 (12)	0.0652 (11)	0.0298 (9)	−0.0045 (11)	0.0042 (10)	−0.0054 (9)
C1	0.0334 (13)	0.0335 (13)	0.0363 (14)	0.0024 (11)	0.0021 (11)	0.0060 (11)
C2	0.0315 (12)	0.0395 (14)	0.0391 (15)	−0.0086 (12)	−0.0029 (11)	0.0020 (12)
C3	0.0277 (12)	0.0404 (13)	0.0429 (15)	−0.0040 (12)	0.0035 (11)	0.0041 (12)
C4	0.0294 (12)	0.0326 (12)	0.0417 (14)	0.0046 (11)	−0.0010 (11)	0.0021 (11)
C5	0.0293 (12)	0.0412 (13)	0.0463 (15)	−0.0009 (12)	−0.0045 (12)	0.0000 (12)
C6	0.0263 (12)	0.0430 (13)	0.0470 (15)	−0.0029 (11)	0.0030 (11)	0.0068 (13)
C7	0.0336 (12)	0.0431 (13)	0.0431 (14)	0.0062 (12)	−0.0021 (12)	−0.0074 (12)
C8	0.0223 (11)	0.0441 (14)	0.0344 (13)	0.0009 (12)	−0.0012 (11)	−0.0054 (12)
C9	0.0334 (13)	0.0521 (14)	0.0356 (14)	0.0060 (12)	0.0028 (11)	−0.0071 (12)
C10	0.0315 (13)	0.0572 (16)	0.0385 (14)	−0.0011 (15)	0.0029 (12)	−0.0075 (14)
C11	0.0277 (12)	0.0460 (14)	0.0303 (12)	0.0012 (13)	−0.0001 (11)	0.0007 (12)
C12	0.085 (2)	0.080 (2)	0.0393 (15)	−0.023 (2)	−0.0078 (16)	0.0111 (16)

### Geometric parameters (Å, °)

**Table:**

O1—C1	1.374 (3)	C4—C5	1.392 (3)
O1—H1	0.8200	C4—C7	1.507 (3)
O2—C8	1.430 (3)	C5—C6	1.382 (3)
O2—H2	0.8200	C5—H5A	0.9300
O3—C11	1.210 (3)	C6—H6A	0.9300
O4—C11	1.320 (3)	C7—C8	1.543 (3)
O4—H4	0.8200	C7—H7A	0.9700
O5—C10	1.200 (3)	C7—H7B	0.9700
O6—C10	1.333 (3)	C8—C11	1.515 (3)
O6—C12	1.446 (3)	C8—C9	1.526 (3)
C1—C2	1.380 (3)	C9—C10	1.494 (3)
C1—C6	1.384 (3)	C9—H9A	0.9700
C2—C3	1.378 (3)	C9—H9B	0.9700
C2—H2B	0.9300	C12—H12A	0.9600
C3—C4	1.380 (3)	C12—H12B	0.9600
C3—H3A	0.9300	C12—H12C	0.9600
			
C1—O1—H1	109.5	C8—C7—H7B	108.5
C8—O2—H2	109.5	H7A—C7—H7B	107.5
C11—O4—H4	109.5	O2—C8—C11	108.40 (17)
C10—O6—C12	116.2 (2)	O2—C8—C9	107.55 (19)
O1—C1—C2	117.2 (2)	C11—C8—C9	112.63 (19)
O1—C1—C6	123.0 (2)	O2—C8—C7	110.03 (18)
C2—C1—C6	119.8 (2)	C11—C8—C7	108.44 (18)
C3—C2—C1	119.5 (2)	C9—C8—C7	109.77 (19)
C3—C2—H2B	120.2	C10—C9—C8	114.4 (2)
C1—C2—H2B	120.2	C10—C9—H9A	108.6
C2—C3—C4	122.3 (2)	C8—C9—H9A	108.6
C2—C3—H3A	118.9	C10—C9—H9B	108.6
C4—C3—H3A	118.9	C8—C9—H9B	108.6
C3—C4—C5	117.2 (2)	H9A—C9—H9B	107.6
C3—C4—C7	121.8 (2)	O5—C10—O6	123.3 (2)
C5—C4—C7	121.0 (2)	O5—C10—C9	125.6 (2)
C6—C5—C4	121.5 (2)	O6—C10—C9	111.1 (2)
C6—C5—H5A	119.2	O3—C11—O4	125.3 (2)
C4—C5—H5A	119.2	O3—C11—C8	123.2 (2)
C5—C6—C1	119.6 (2)	O4—C11—C8	111.43 (19)
C5—C6—H6A	120.2	O6—C12—H12A	109.5
C1—C6—H6A	120.2	O6—C12—H12B	109.5
C4—C7—C8	115.19 (19)	H12A—C12—H12B	109.5
C4—C7—H7A	108.5	O6—C12—H12C	109.5
C8—C7—H7A	108.5	H12A—C12—H12C	109.5
C4—C7—H7B	108.5	H12B—C12—H12C	109.5
			
O1—C1—C2—C3	179.3 (2)	C4—C7—C8—C9	−174.1 (2)
C6—C1—C2—C3	0.3 (4)	O2—C8—C9—C10	−68.8 (2)
C1—C2—C3—C4	−0.2 (4)	C11—C8—C9—C10	50.5 (3)
C2—C3—C4—C5	0.1 (3)	C7—C8—C9—C10	171.5 (2)
C2—C3—C4—C7	−178.7 (2)	C12—O6—C10—O5	−2.1 (4)
C3—C4—C5—C6	0.0 (3)	C12—O6—C10—C9	176.6 (2)
C7—C4—C5—C6	178.7 (2)	C8—C9—C10—O5	−5.7 (4)
C4—C5—C6—C1	0.1 (4)	C8—C9—C10—O6	175.6 (2)
O1—C1—C6—C5	−179.2 (2)	O2—C8—C11—O3	−13.9 (3)
C2—C1—C6—C5	−0.2 (4)	C9—C8—C11—O3	−132.8 (2)
C3—C4—C7—C8	−78.5 (3)	C7—C8—C11—O3	105.5 (2)
C5—C4—C7—C8	102.8 (3)	O2—C8—C11—O4	169.74 (19)
C4—C7—C8—O2	67.7 (3)	C9—C8—C11—O4	50.9 (3)
C4—C7—C8—C11	−50.7 (3)	C7—C8—C11—O4	−70.8 (2)

### Hydrogen-bond geometry (Å, °)

**Table:**

*D*—H···*A*	*D*—H	H···*A*	*D*···*A*	*D*—H···*A*
O1—H1···O3^i^	0.82	1.96	2.775 (2)	172
O2—H2···O1^ii^	0.82	2.34	2.888 (2)	125
O4—H4···O2^iii^	0.82	1.85	2.639 (2)	161
C12—H12B···O5^iv^	0.96	2.42	3.268 (4)	148

Symmetry codes: (i) −*x*, *y*−1/2, −*z*+1/2; (ii) −*x*+1, *y*+1/2, −*z*+1/2; (iii) *x*−1, *y*, *z*; (iv) *x*+1/2, −*y*+3/2, −*z*.

## References

[bb1] Bruker (1998). *SMART* and *SAINT* Bruker AXS Inc., Madison, Wisconsin, USA.

[bb2] Heller, W. & Tamm, C. (1974). *Helv. Chim. Acta*, **57**, 1766–1784.

[bb3] Jiang, J. Q., Li, Y. F., Chen, Z., Min, Z. D. & Lou, F. C. (2006). *Steroids*, **71**, 1073–1077. 10.1016/j.steroids.2006.09.005 17112557

[bb4] Jiang, J. Q., Ye, W. C., Chen, Z., Lou, F. C. & Min, Z. D. (2002). *J. Chin. Pharm. Sci.* **11**, 1–3.

[bb5] Sheldrick, G. M. (2008). *Acta Cryst.* A**64**, 112–122. 10.1107/S0108767307043930 18156677

[bb6] Siemens (1998). *XP* Siemens Analytical X-ray Instruments Inc., Madison, Wisconsin, USA.

